# How does a wetland plant respond to increasing temperature along a latitudinal gradient?

**DOI:** 10.1002/ece3.8303

**Published:** 2021-11-03

**Authors:** Regina Lindborg, Matti Ermold, Lenka Kuglerová, Roland Jansson, Keith W. Larson, Ann Milbau, Sara A. O. Cousins

**Affiliations:** ^1^ Landscape, Environment and Geomatics Department of Physical Geography Stockholm University Stockholm Sweden; ^2^ Bolin Centre for Climate Research Stockholm University Stockholm Sweden; ^3^ Department of Forest Ecology and Management Swedish University of Agricultural Sciences Umeå Sweden; ^4^ Department of Ecology and Environmental Science Umeå University Umeå Sweden; ^5^ Climate Impacts Research Centre Department of Ecology and Environmental Science Umeå University Umeå Sweden; ^6^ Province of Antwerp Department of Sustainable Environment and Nature Policy Antwerp Belgium

**Keywords:** *Caltha palustris*, climate change, experiment, open top chamber, traits, wetland

## Abstract

Global warming affects plant fitness through changes in functional traits and thereby ecosystem function. Wetlands are declining worldwide, and hence, ecosystem functions linked to wetlands are threatened. We use *Caltha palustris* “a common wetland plant” to study whether warming affects growth and reproduction differently depending on origin of source population, potentially affecting phenotypic response to local climate. We conducted a 2‐year *in situ* temperature manipulation experiment using clone pairs of *C*. *palustris* in four regions, along a 1300‐km latitudinal gradient of Sweden. Open‐top chambers were used to passively increase temperature, paired with controls. Growth and reproductive traits were measured from 320 plants (four regions × five sites × two treatments × eight plants) over two consecutive seasons to assess the effect of warming over time. We found that warming increased plant height, leaf area, number of leaves, and roots. High‐latitude populations responded more strongly to warming than low‐latitude populations, especially by increasing leaf area. Warming increased number of flowers in general, but only in the second year, while number of fruits increased in low‐latitude populations the first year. Prolonged warming leads to an increase in both number of leaves and flowers over time. While reproduction shows varying and regional responses to warming, impacts on plant growth, especially in high‐latitude populations, have more profound effects. Such effects could lead to changes in plant community composition with increased abundance of fast‐growing plants with larger leaves and more clones, affecting plant competition and ecological functions such as decomposition and nutrient retention. Effects of warming were highly context dependent; thus, we encourage further use of warming experiments to predict changes in growth, reproduction, and community composition across wetland types and climate gradients targeting different plant forms.

## Graphical abstract

In a warming experiment in wetlands across 1300‐km latitudinal gradient, we found that clonal pairs of Caltha palustris increased in plant height, leaf area, number of leaves, and roots. High‐latitude populations responded more strongly than low‐latitude populations. Increasing temperatures could lead to changes in plant community composition with increased abundance of fast‐growing plants with larger leaves and more clones, affecting plant competition as well as services such as flood attenuation and nutrient retention.

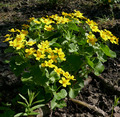

## 1 INTRODUCTION

Global warming is considered one of the major drivers of environmental change, threatening biodiversity, ecosystem function, and ultimately the delivery of ecosystem services. This has already resulted in the migration of species both toward higher latitude and altitude (Chen et al., 2011; Parolo & Rossi, 2008), but also to changes in vegetation and the extinction of populations in their native habitat (Thuiller et al., 2005; Walther et al., 2002). Especially, plant communities at high latitudes are sensitive to warming (Bjorkman et al., 2020), which may strongly affect plants by delaying their winter preparation, prematurely interrupting dormancy through warm spells, and damaged plant tissue due to lack of snow cover (Taulavouri, 2013). Further, changes in climatic conditions have been shown to favor invasive species leading to new community structures for both plants and animals (Walther et al., 2009), ultimately changing ecosystem function and service generation (Dossena et al., 2012).

Climate warming effects on vegetation play out differently depending on plant species, plant community, and plant traits (de Bello et al., 2010). Warming alters primary production, mainly through functional traits related to growth and reproduction of plants (Baruah et al., 2017; Diaz et al., 2004). Changes in phenology, such as timing of budburst or flowering, are one of the major plant responses to climate change (Menzel et al., 2006; Peñuelas & Filella, 2001). However, not all species or functional groups of plants respond in the same way to changes in temperature. For example, experimental work by Wolkovich et al. (2012) and Kudo et al. (2008) found that plant species that flower early in the spring respond more quickly, and potentially even adapt better to warming in comparison with late summer flowering species. Further, a long‐term study showed increased plant reproduction after several years of exposure to elevated temperature (Arft et al., 1999). On the contrary, Anderson (2016) found decreases in individual reproductive output as a direct response to warming. Long‐term studies on the effect of climate warming found that flowering and bud burst advanced by 6 days since the 1980s, while leaf abscission was delayed by almost 5 days, leading to an overall lengthening of the growing season (Cleland et al., 2007; Menzel & Fabian, 1999). Investigating changes in growth and reproduction in plant community as well as for single species as a response to climate will enhance predicting changes in growth, reproduction, and community composition in different habitats and ecosystems and for different plant forms. Such knowledge could underpin a better insight into changes in ecosystem function and also services (Balvanera et al., 2006; Cardinale et al., 2012); for example, in wetlands, clonality and number of shoots may affect flow regulation through water holding capacity, and specific leaf area (SLA) may though decomposition and accumulation affect greenhouse gas emission and nutrient retention (Moor et al., 2017).

Wetlands are ecosystems of great importance for many essential services, such as storage of carbon, filtering, and provision of drinking water and are also important for biodiversity (Mitsch et al., 2015; Mitsch & Gosselink, 2000; Zedler & Kercher, 2005). Being at the interface of terrestrial and aquatic ecosystems, wetland vegetation and related plant functional traits may predict changes in ecosystem function and services (Díaz & Cabido, 2001; Lavorel & Garnier, 2002), important for water, nutrient, and carbon cycling. Wetlands are strongly affected by climate change (Dixon et al., 2016; Junk et al., 2013; Ramsar Convention, 2018), leading to both direct and indirect effects on wetland hydrology (direct) and species composition (indirect) (Brinson & Malvárez, 2002; Junk et al., 2013; Short et al., 2016). Type of wetland, for example, bog, swamp, marsh, riparian zone, or fen, determines whether water covers the soil or is at or near its surface, either year‐round or seasonally strongly affecting their ecological functions (Moor et al., 2017). Especially, riparian zones, used in this study, are regularly disturbed by flooding and their reaction to warming in the timing, magnitude, and duration of floods differ compared with typical wetlands where subsurface hydrology plays a larger role than surface hydrology (Rivaes et al., 2013; Ström et al., 2012).

The limited number of studies, as well as the range of effects on wetland plant species traits and community responses to climate warming, inhibits our ability to fully understand how ecosystems function and services will be affected. Breeuwer et al. (2008) found an increase in plant height for *Sphagnum* species, but a similar response was not found for salt marsh species (Charles & Dukes, 2009). Studies of leaf area on two boreal wetland plants found no or even negative effects of warming (Zou et al., 2014), whereas Moor et al. (2015) found that warming increased the abundance of fast‐growing plants with larger leaves, a difference that could affect key ecosystem services such as flood attenuation and short‐term nutrient retention. The relative abundance of growth forms and effects of increased temperature also differed depending on wetland type where increasing temperature in peatlands favored graminoid growth, while shrub cover increased in bogs (Dieleman & Branfireun, 2015; Weltzin et al., 2003). This highlights the difficulty to generalize the potential impact of warming across wetland types, plant groups, and plant traits.

To study the effects of warming on plant communities in situ, three different approaches are commonly used (i) passive warming at the vegetation site by using open‐top chamber (OTC) or active warming by cables, (ii) space for time approach by observing species either at different latitudes or at altitudes, or (iii) reciprocal transplant experiments which move plants to a new location under novel climatic conditions (de Frenne et al., 2011). The use of passive warming facilities to study a naturally occurring warming effect on plant traits can be a useful tool to understand the effect of local climate on phenotypic responses if included in a large latitudinal gradient experimental setup. Since delayed responses to warming have been shown at high latitudes (Arft et al., 1999), studying warming effects over time is also important to fully understand effects of yearly variation. Studying warming through all seasons, especially winter, is also rare (Sanders‐DeMott & Templer, 2017), which make it difficult to understand the full effect of differences among seasons, especially over time.

To investigate the potential effect of increasing temperature on wetlands and to make predictions about change in ecosystem functions, we carried out a field experiment in four riparian wetland regions using open‐top chambers for passive warming and clones (ramets) of the common wetland plant *Caltha palustris* along a 1300‐km latitudinal gradient in Sweden. We measured plant traits related to growth and reproduction to answer the following questions. (i) Does warming by means of OTCs lead to changes in growth (measured as plant height, leaf area, and the number of roots and leaves per plant) and reproduction (number of flowers, fruits, and clones per plant)? (ii) Is warming affecting growth and reproduction differently depending on latitudinal position? (iii) Does extended warming over time lead to compound effects in growth and reproduction (e.g., are plants in the second year of warming significantly larger than plants that received warming only for 1 year?)? We hypothesized that warming will increase growth in traits related to both plant height and leaf area, together with increases in reproductive traits, such as number of flowers and number of fruits. We also hypothesized that the magnitude of the effect will be more pronounced for plants in northern latitudes, since temperature‐related constraints on growth and reproduction will be eased and that warming effects will increase cumulatively over time.

## 2 METHODS

### 2.1 Study species

To evaluate the potential effects of increasing temperature on wetland communities and their ecosystem function, we chose an early flowering species from the family Ranunculaceae, the common wetland plant *Caltha palustris*. It is a perennial herb, 10–40 cm tall and occurs over the whole of Sweden, having a circumboreal distribution ranging from 40° to 75°N and is also found in the Southern Hemisphere. Marshes or other wetlands are the predominant habitat (Schuettpelz & Hoot, 2004). Flowering time ranges from April to May in the Northern Hemisphere depending on altitude and latitude. It can reproduce both clonally and via seeds and is self‐incompatible (Lundqvist, 1992). *Caltha palustris* is pollinated by a wide variety of insects (Judd, 1964), and seeds are usually water dispersed with each flower carrying between 10 and 100 seeds (Clapham et al., 1990).

### 2.2 Experimental design

**FIGURE 1:**
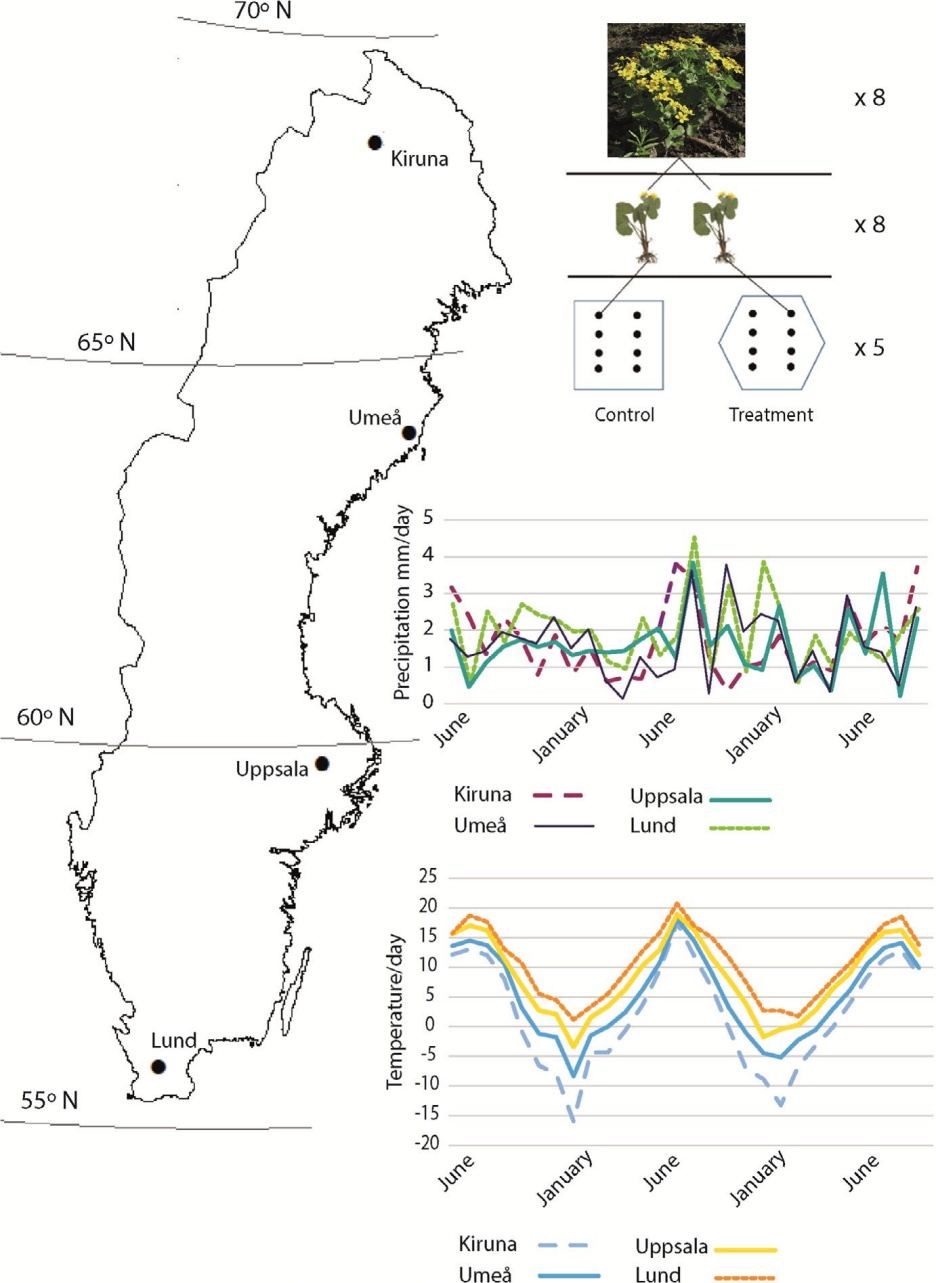
Location of the regions used in the study; Lund, Uppsala, Umeå and Kiruna. To the right a sketch of the experimental setup. One plant of *Caltha palustris* was dug up was split into its ramets, creating clone pairs, whereupon one randomly selected ramet was planted into a control plot and another ramet was planted in the open‐top chamber (warming treatment). This was repeated to leave 8 plant individuals in each treatment and at 5 sites per region. The figure includes monthly precipitation (mm) and average daily temperature for each region during the experiment downloaded and compiled from SMHI (Swedish Metrological and Hydrological Institute)

We conducted a temperature manipulation experiment using open‐top chambers (hereafter OTC) in 20 wetland sites in four regions (five sites in each region) of Sweden, spanning a latitudinal gradient of 1300 km (Figure 1). The OTCs were made of Plexiglas, which approximately transmit 90% of visible light. The open‐top chambers in the experiment were constructed according to the International Tundra Experiment (Arft et al., 1999) with a hexagonal design covering approximately 4.4 m^2^, an opening of 1.5 m^2^, and a height of 0.8 m. Snow and rain can enter the OTCs but there are other side effects besides increasing temperature, for example, less wind, increased relative humidity, and CO_2_ concentration (Dabros et al., 2010). OTC is still the most commonly used method to passively increase the temperature in natural experiments.

Sites in Lund, Uppsala, and Umeå were situated in the riparian zone of 1st‐ to 3rd‐order streams and therefore had similar hydrology with shading provided by deciduous trees (Lund and Uppsala) and coniferous trees (Umeå). In the northernmost region, Kiruna, all sites had little to no shading provided by trees. The OTC was placed close ca 3–4 m from the stream banks in all sites except in Kiruna where they were placed 3–4 m from the mainstream flow in the wetland. The OTCs were kept in place over the whole course of the 2‐year experiment.

In the summer of 2013, we dug up eight adult individuals of *C*. *palustris* from each site to be included in the experiment. The plants were then carefully divided into ramets (hereafter clones) with as little damage as possible to above‐ and belowground parts. *Caltha palustris* forms short‐lived rhizome (epigeogenous rhizomes) and rarely also stolons (not found in this study) (Klimešová et al., 2017). Therefore, clone was defined as a as a rooting unit, a ramet, connected by short rhizome. Two randomly selected clones from each plant were chosen with one being replanted into the OTC treatment, while the other clone was placed outside the warming facility in ambient temperature and thus acted as a control. Within each OTC and the control, the clones were spatially arranged in lines of two by four and spaced at equal distance from each other, approx. 30 cm, to avoid plant–plant interactions during the experiment (Figure 1). The total number of samples was 320 (4 regions × 5 sites × 8 replicates × 2 treatments).

At each site, we placed temperature loggers 10 cm above and 5 cm below in the soil surface within the OTC and in the control site. The loggers, brand iButton Thermocron with resolution of 0.5°C, recorded temperature every second hour and were covered by a plastic sun shield. We calculated both the mean air and the soil temperature for the entire experimental period, spring temperature, and the mean growing season temperature, as well as growing degree hours above 5°C for the period of January 1 to June 15. Growing season was defined as when mean temperature is above 5°C during 24 h. For comparison, we also compiled monthly precipitation and temperature data (open source SMHI) from the most adjacent whether station for all four sites.

### 2.3 Measured traits

We selected four traits related to growth: plant height, leaf area, number of leaves, and number of roots, and three traits related to reproduction: number of flowers, number of fruits, and number of clones. All traits were measured during two consecutive growing seasons in 2014 and 2015. Traits were measured three times, once in the spring 2014 and in 2015 at the peak of the flowering as well as during peak biomass and fruit production in the summer of 2015 (see Tables S1 and S2 for sampling dates and temperature data). Root number and the number of clones were measured at the end of the experiment in September (Kiruna and Umeå) and October (Lund and Uppsala) 2015. The date of the spring sampling of *C*. *palustris* in Lund was based on phenological reports of flowering reported by citizens to the Swedish National Phenological Network (www.naturenskalender.se) and by personal observation in the actual regions close to the experiment for Uppsala, Umeå, and Kiruna. Plant height, number of leaves, and leaf area were measured non‐destructively on all three sampling occasions. Plant height was assessed for each individual by measuring the length of the longest petiole, which always exceeded the length of the stem. Leaf area was assessed by taking photographs of the leaves against graph‐paper background and later analyzed using ImageJ (Schneider et al., 2012). Leaf number was counted as the total number of leaves per individual including number of damaged or clearly missing leaves. At the end of the experiment in the autumn of 2015, we counted the total number of storage roots (dead or alive, excluding fine roots >2–3 mm) per plant.

To analyze reproductive traits, we counted the number of clones per individual at the end of the experiment as the number of additional clones compared with the number initially planted. Number of fruits was also measured once as the sum of fruits, including damaged or clearly missing fruits in the summer of 2014, whereas number of flowers was measured as the sum of open and closed flowers in both spring 2014 and 2015, which is the time peak flowering for *C*. *palustris*. All location, temperature, and trait data are available on Dryad (full reference TBA on acceptance).

### 2.4 Data analysis

To examine the effect of experimental warming on plant height and leaf area, we used a generalized linear mixed effect (GLMM model) with either plant height or leaf area as response variable, and region (Lund, Uppsala, Umeå, and Kiruna), treatment (Ambient and OTC), and time of observation (Spring 2014, Summer 2014, and Spring 2015) as explanatory variables. We further included clones nested within site as a random effect to account both for spatial autocorrelation between sites and for the genetic relatedness of the plant individuals in our study. The data for both plant height and leaf area were transformed using natural logarithm to achieve normally distributed residuals. Best model fit was assessed by starting with the full model (main factors and interactions) and then dropping subsequent non‐significant terms while comparing the fit of the models to the data using a likelihood ratio test until either only significant terms were included in the model or until dropping terms did not yield a significantly better model. The number of leaves per individual was analyzed using generalized linear mixed effect models with a Poisson distribution and a log‐link function using the same predictor variables as above (region, treatment, and time). Similarly, the number of roots was also analyzed using Poisson error distribution and region and treatment as predictor variables. The predictor time was not included as root number was only assessed at the end of the experiment.

To assess the effect of warming on the reproductive traits of both flower and fruit number, we used generalized linear mixed effect models (GLMM) using negative binomial distribution. Models on flower number included all predictor variables (region, treatment, and time) for the analysis while fruit number only included region and treatment. Clonal production was relatively low with most individuals producing either one, a maximum of three, or no clonal offspring. Therefore, we recoded the number of clonal offspring to a binary variable as either present or absent (Yes or No) which we then analyzed using a binomial distribution with logit link function with treatment and region as predictor variables. Here again, we included site and clone as random factors with clone nested in site for all analysis on reproduction.

Since the sampling time for each region could not be standardized on a common metric (growing degree hours or day of first flower), we analyzed data on growth and reproduction for each region and each time point separately as well. Thus, we used the same model structure outlined above, excluding region as predictor variable but instead conducting separate analyses per region (Lund, Uppsala, Umeå, and Kiruna) and time, only including treatment as predictor variable. All models were checked graphically if they fulfill the assumptions of normality and homoscedasticity of residuals. All statistical analyses were carried out using R (Rstudio) (R Development core team, 2018) and the *lme4* (Bates & Maechler, 2009) and *glmm* (Knudson et al., 2020) libraries. To simplify graphical visualization, we plotted relative trait value changes between control and treatment rather than absolute values for both since we were not interested in trait difference due to latitudinal variation in temperature or other variables.

## 3 RESULTS

The effect of warming differed between region, year, and location (Table S1). Warming increased average air temperature by 0.2° and 0.1°C in Lund and Kiruna, respectively, while air temperature did not change for Uppsala and even decreased by 0.4°C for Umeå. The decrease in the mean temperature for Umeå was mainly attributed to an unexpected cooling effect of the chambers in the winter of 2013–14, due to lack of snow cover. Average soil temperature increased by 0.1° and 0.2°C for Lund and Umeå, respectively, while it remained unaffected for Uppsala and decreased for Kiruna (0.4°C). The sum of growing degree hours (hereafter GDH) for air temperature and for the first season (2014) showed a moderate increase in all but one region (Kiruna). GDH in the soil increased in Lund and Umeå while it decreased in Uppsala. OTCs increased mean temperatures of the growing season for several regions while it also increased maximum temperatures in all regions except Umeå.

**FIGURE 2:**
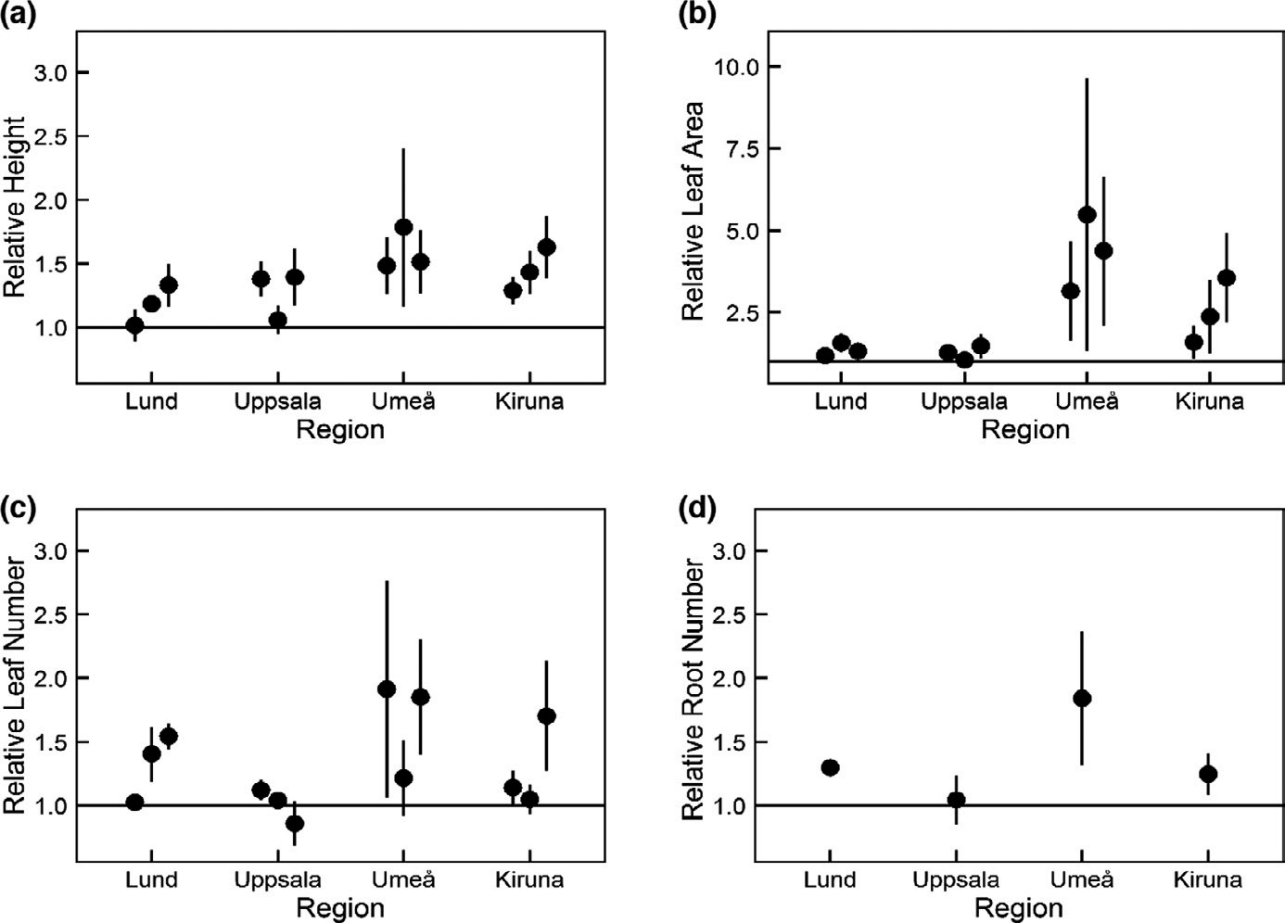
Effects of experimental warming on clone pairs of Caltha palustris (*N* = 40) in four different regions over time on (a) relative plant height, (b) relative leaf area, (c) relative number of leaves and d) relative number of roots (means ± SE) measured during 3 years (a–c) and 1 year (d). Horizontal line at *y* = 1 represents no change between clonal pairs

Although the temperature did not increase in all OTCs during the whole period and for all regions (Table S1), the OTC treatment had a significant effect on many of the selected traits on the clone pairs (Table 1). Plant height increased significantly in all OTC treatments in all regions and was on average 1.7cm taller than in plants from the control (Figure 2). However, plant height did not increase uniformly across regions. Plant height in the OCTs increased the most in the two northernmost regions, 1.5‐fold in Umeå and 1.6‐fold in Kiruna, compared with a 1.2‐fold increase for treatment plants from the southern regions (Figure 2). In Lund, the plant height did not change in spring 2014, and in Uppsala, plant height increased only in the spring of both 2014 and 2015 while no effect could be found in the summer (Figure 2 and Table S1). For absolute mean values for the trait measures over time per region, see Table S2.

**TABLE 1:** Results of the linear mixed effect model on the effect of warming and time on growth and reproduction on 160 clone pairs of *Caltha palustris*

	Warming	Time	Region	Warming × Time	Warming × Region	Time × Region	Warming × Time × Region
Growth							
Plant height^a^	↑6.1*	↕5*	↕12.3**	ns	ns	39.1***	ns
Leaf area^a^	↑6.7**	↕12.7**	ns	ns	ns	72.9***	ns
No. of leaves^c^	↑8.8**	↕39.3***	ns	ns	9.6**	181***	16.6***
No. of roots^b^	ns	–	ns	–	ns	–	–
Reproduction							
No. of flowers^d^	↑7.4**	↑33.8***	ns	ns	7.8*	144.6***	8.6*
No. of fruits^d^	Ns	–	↑11.1**	–	11.6**	–	–
No. of clones^e^	Ns	–	↕6.3*	–	5.2⁽*⁾	–	–

Note

The numbers refer to effect size. ↓, decrease; ↑, Increase; ↕, non‐directional; *, *p* < .01; **, *p* < .001; ***, *p* < .0001; ⁽*⁾, *p* < .1; Ns, not sig.; –, not applicable.

^a^ Log transformed.

^b^ sqrt transformed.

^c^ Poisson error distribution used for data.

^d^ Negative binomial error distribution used for data.

^e^ Binomial error distribution used for data.

Similar to plant height, leaf area increased in the OTCs (Table 1). The treated plants showed a more than 10 percent increase in leaf area in comparison with the controls. In general, warming had the largest effect on leaf area in the second year of the experiment (Table 1 and Figure 2). The increase in leaf area showed regional differences, where relative leaf area increased more in plants in the northernmost regions in comparison with plants from the southern regions. Warming increased leaf area already in the first year of warming in Umeå and Lund, while it only showed an effect on plants from Uppsala and Kiruna in the second growing season. Number of leaves per plant also increased under experimental warming with an average of 6.8 leaves observed in the treatments in comparison with 6.1 leaves in the ambient control (data not shown). Similar to the observation for leaf area, number of leaves increased in the treatment plants from Lund, Umeå, and Kiruna, while a negative effect of warming was found for plants from Uppsala (Figure 2). However, the number of leaves only showed a significant response to warming in the spring of 2015 (Table S2). Also, the number of roots increased under warming in Lund and Umeå (Figure 2).

**FIGURE 3:**
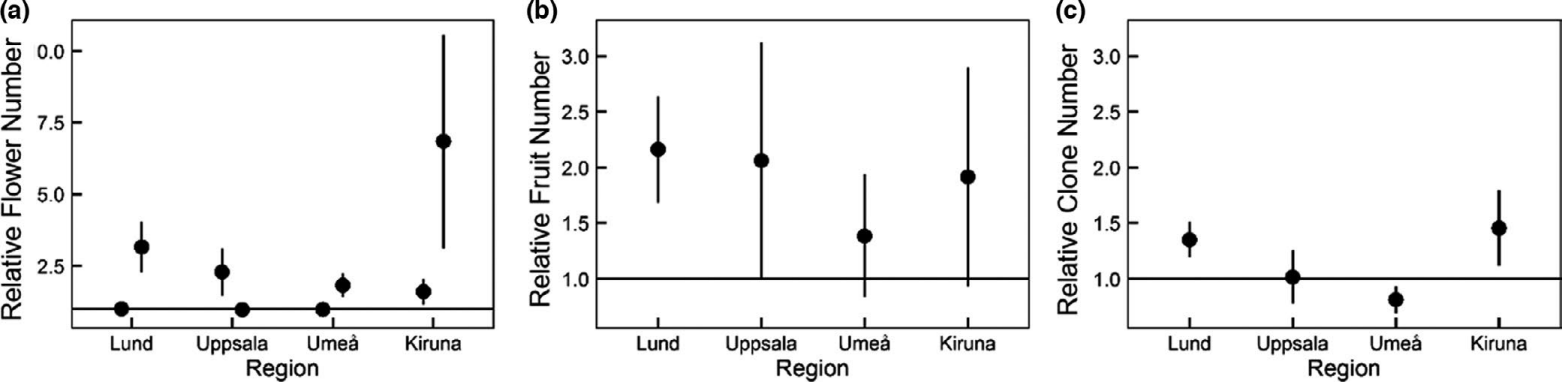
Effects of experimental warming on clone pairs of Caltha palustris (*N* = 40) in four different regions over time on (a) relative flower number (b) relative number of fruits and (c) relative number of clonal offspring (means ± SE), measured during 2 years (a) and 1 year (b–c). Horizontal line at *y* = 1 represents no change between clonal pairs

Number of flowers increased by 50 percent under warming with significant differences observed between regions (Table 1). Flower number increased in the second year in Lund and Kiruna, but not in the other two regions (Table S2 and Figure 3). Generally, the number of fruits was not significantly affected by warming, but the average fruit production was slightly higher in all regions when warmed (Table 1 and Figure 3). The number of clones was also not affected by warming (Table 1 and Figure 3). However, in interaction with region, the number of clones tended to increase with warming in Lund but were higher in the ambient control for plants in Umeå.

## 4 DISCUSSION

By dividing plants into clones, we could measure the individual trait response to experimentally increased temperatures using open‐top chambers (OTC). In general, we found that the OTC treatment led to an increase in all growth‐related traits (plant height, leaf area, leaf number, and number of roots) but only significantly affected one of the three traits associated with reproduction, namely the number of flowers. The effect of the treatment also increased with time for most of these traits and regions, except for Uppsala that showed a diverging pattern. The interaction between region and warming generally had a stronger effect on reproductive traits compared with growth‐related traits, indicating that other factors than temperature are important for these traits.

The positive effect of warming on growth traits has been shown in earlier studies, either in experimental settings (de Frenne et al., 2011; Hudson et al., 2011; Walker et al., 2006) or when modeling trait data under current and future climate (Moles et al., 2014; Moor et al., 2015). We found that increasing temperature had the strongest effect on plant height, which was detected already after 1 year of warming in all but one region (Lund). This was probably an effect of the relatively cold spring in that region in 2014, confirmed by analysis of mean temperature (c. 2 degrees lower than average) and growing degrees hours (GDH) at the time of sampling. Although we can only speculate, we probably would have seen an effect on both plant height and leaf area if we conducted our sampling in Lund later in spring. Winter temperatures can also be important for plants under climate change. Kreyling et al. (2019) showed that winter warming may increase soil respiration more than summer warming, prolonging growing seasons and increasing aboveground biomass production (which in turn might interrupt dormancy and damage tissue in Spring and Autumn (Taulavouri, 2013). However, in colder regions at high latitudes in our experiment in northern Sweden, the vegetation is dormant during the coldest months and we could not find any damaged plant tissue related to Spring or Autumn events. However, one important difference to previously warming experiment that have primarily been carried out in alpine regions, in grasslands or in forests, is that the surface water in wetlands, can rapidly thaw a snow cover but also keep the site cool during warmer periods.

As hypothesized, leaf area also increased under warming which is congruent with recent literature (Hudson et al., 2011; Moor et al., 2015). However, plants from the northernmost region (Kiruna) increased their leaf area significantly only in the last year of the study, while plants from Lund and Umeå responded with increasing leaf area already in the first experimental year. Despite that, we found an overall larger mean increase in leaf area for both northern regions compared with the southern regions. This suggests a stronger effect on plant growth in *C*. *palustris* at its northern edge of the geographic range, which potentially will allow this species to colonize new habitats and migrate further north. Cumulative warming effects could not be clearly shown in our results, as there were differences among traits and regions (Figure 2), although there were some indications of cumulative warming in both Lund and Kiruna.

Interestingly, we still found effects of the OTC treatment despite the low increase in mean temperature and GDH. Studies by de Frenne et al. (2011) and Hudson et al. (2011) reported an increase in mean temperature of 1–2 degree Celsius or increases in GDH by ten to twenty percent in OTCs compared with the surrounding, which is far greater than the warming achieved in our experiment. This is most probably because these studies were performed in deciduous forests and on the Arctic tundra with drier soils with less moving groundwater compared with wetlands. This suggests that even small to moderate increases in mean temperature can lead to rapid change in growth and reproduction. This trend has also been found when studying changes in plant biomass under warming (biomass increase) for seed bank communities (Baldwin et al., 2014) from different latitudes.

We also found an increase in the number of leaves toward the end of the experiment in some but not all regions, which was most likely coupled with an increase in number of flowers. Both fast growth and early flowering could be related to overwintering buds (Schnablová et al., 2021). Although not specifically tested in this study, pre‐formed buds could potentially explain the increase in both flower and number of leaves for individual plants, as pre‐formed buds have been documented for *C*. *palustris* (Schnablová et al., 2021). Number of leaves are seldom assessed in studies concerning warming, but might be beneficial to include in future studies in wetlands. This is especially useful when drawing conclusions about ecological functions, since also aboveground plant structure has been shown to influence soil erosion and flood attenuation (Burylo, Rey, & Amathys, 2012; Burylo, Rey, Bochet, et al., 2012). In addition to an increase in leaf number, we also found an effect in the number of roots following warming, particularly in Lund and Umeå. This might be explained by the proportionally higher warming effect in the soil for those regions. Freeze‐thaw events during winter are unlikely in Kiruna and Umeå and the plants are dormant during the coldest months, but freeze‐thaw events could potentially be important in southern riparian zones during the coldest months as the temperature fluctuates around zero centigrades and where shallow groundwater can cause severe root damages if it freezes.

Delayed response of reproduction to warming has been shown in the Arctic tundra, where reproduction only increased after several years of warming (Arft et al., 1999). We found a similar response in our experiment as reproduction in terms of flower number only increased in the second year. On the contrary, number of fruits increased already the first year for plants in the southernmost region. Unfortunately, we cannot say whether this increase in the number of fruits stems from individuals with more flowers in the spring before transplantation. However, OTCs are generally known to disrupt plant–pollinator interactions by decreasing insect density and visitation, which may have negative effects on flower visitation rates and thereby fruit set (de Frenne et al., 2010, 2011). We therefore speculate that the effect on number of fruits is likely to be related to warming at that site. Interestingly, higher flower number also mirrored the increase in number of leaves observed in the second year of the experiment. This might indicate that an increase in reproductive output (flowers) requires an increase in photosynthetic capacity by increasing either number of leaves or leaf area. A few studies (Craufurd et al., 2000; Ehlers & Olesen, 2004) found an increase of flower number with leaf number perhaps giving support for this correlation.

Uppsala (mid‐southern region) showed somewhat diverging patterns concerning plant height and leaf area compared with the three other regions. It has been shown that OTCs could change several sheltering parameters like decreasing effects of surface flooding, snow, and wind depending on forest cover (De Long et al., 2016). We noted a high growth (in abundance and height) of other plants in the chamber at the time of sampling (not measured), which we did not observe in the OTCs in the other regions. It is possible that more competitive plants which start to grow later in the season may potentially outgrow *C*. *palustris* and thereby decrease the availability of light and soil nutrients, which in turn may lead to the observed differences despite higher temperatures. This is partly confirmed by de Frenne et al. (2010) showing that species composition changed toward faster growing species under a more open canopy. Warming effects on *C*. *palustris* in Uppsala could therefore be more pronounced with the slightly open canopy, favoring a higher abundance of fast‐growing competitive species compared with the other sites.

Transferring results from single species experiments to effects on wetland plant communities is important to understand the full effect of climate change, but it is also difficult as species and functional groups might respond differently to warming (de Frenne et al., 2011; Schwarzer et al., 2013), as well as to changes in precipitation and evapotranspiration (Roth et al., 2021). However, using *C*. *palustris* as a wetland representative, we would expect an increase in growth of many of the earlier flowering species, which together with changes in reproductive output could lead to changes in community composition. In contrast, our results also suggest that warming response of earlier flowering wetland plants might be offset in the summer in productive and more open wetlands, leading to a reduction in growth and reproduction for early flowering plants depending on wetland type and local setting. Although not detected in our study, cumulative warming effect on plant traits might increase the future competitiveness in early flowering wetland species. Thus, measuring response of early flowering plant species can be fundamental to assess vulnerability of wetlands under climate change (Dullinger et al., 2012). Another crucial factor for predicting changes in wetland plants to warming relates to hydrological parameters (soil moisture, precipitation, and flood peaks), which have been shown to influence growth and reproduction (Jansson et al., 2019; Weltzin et al., 2000).

In terms of ecosystem function, warming is predicted to increase primary productivity (especially in the north) which would increase the short‐term storage of carbon and nitrogen in plant tissue. Root number also increased with warming, which could help increase plant ability to store carbon (Diaz & Cabido, 1997). Although the increase in growth‐related traits was not uniform across all regions, the detected increases in plant height and leaf area also suggest a future increase in sediment retention and flood attenuation which could prove beneficial under the projected increase in severity and frequency of extreme flooding events. To link specific plant traits to key ecological functions is therefore needed to understand and predict effects on plant communities caused by climate change (Burylo, Rey, & Amathys, 2012; Burylo, Rey, Bochet, et al., 2012; Heilmeier, 2019; Moor et al., 2017). Based on this study, we encourage to further explore the use of OTCs in seasonal warming experiments, including measuring other kinds of sheltering variables, to predict changes in growth, reproduction, and community composition in different wetland types and for different plant forms (early growth vs. late growth, herbs vs grasses).

## AUTHOR CONTRIBUTIONS

**Regina Lindborg:** Writing–original draft (equal); writing–review and editing (equal). **Matti Ermold:** Conceptualization (lead); data curation (lead); formal analysis (lead); investigation (lead); methodology (lead); writing–original draft (supporting). **Lenka Kuglerová:** Data curation (supporting); writing–review and editing (supporting). **Roland Jansson:** Data curation (supporting); writing–original draft (supporting). **Keith W. Larson:** Data curation (supporting); writing–original draft (supporting). **Ann Milbau:** Data curation (supporting); writing–original draft (supporting). **Sara A. O. Cousins:** Conceptualization (equal); funding acquisition (lead); project administration (lead); resources (lead); supervision (lead); writing–original draft (equal); writing–review and editing (equal).

## Supporting information

Table S1‐S2

## Data Availability

Upon acceptance data on sampling locations, dates, climate data, and trait measurements will be uploaded to Dryad.
