# Supplementary material for: How does a wetland plant respond to increasing temperature along a latitudinal gradient?
Source: Ecol Evol. 2021 Nov 3;11(22):16228–38. doi: 10.1002/ece3.8303 (PMC8601882; doi:10.1002/ece3.8303)
Supplement: Supplementary file 1 — Table S1‐S2 [file ECE3-11-16228-s001.docx]

Supplementary material

Table 1: Results of the temperature logger data over the entire experiment period. The experiment was conducted at four sites along an 1300 km long gradient. At each site there were Open Top Chambers elevating temperature and controls (ambient temperature). T below = Temperature at 5 cm below soil surface, T above = Temperature at 10 cm above the soil surface No value (-) indicate missing values.

Table 2: Absolute values for trait measurements based on 80 plants Caltha palustris per region. The experiment was conducted at four regions along an 1300 km long gradient from south to north of Sweden. At each region there were five sites of Open Top Chambers elevating temperature and controls (ambient temperature). Traits were measured during the peak flowering PF or at the peak biomass PM within each region. Root number was measured at the end of the project when plants were dug up and number of clones. The table presents the absolute values as mean and standard deviation (SD) for each region.

|  |  | Height in cm ± SD | | Leaf area in cm² ± SD | | Leaf number ± SD | | Root number ± SD | |
| --- | --- | --- | --- | --- | --- | --- | --- | --- | --- |
| Region | Time / Year | ambient | elevated | ambient | elevated | ambient | elevated | ambient | elevated |
| Lund | PF 2014 | 5.35 ± 1.6 | 5 ± 1.3 | 8.3 ± 3.5 | 8.9 ± 3.3 | 5.3 ± 1.5 | 5.4 ± 1.6 | - | - |
| Lund | PF 2015 | 22.5 ± 6.3 | 25.3 ± 6.5 | 63.5 ± 27.6 | 71.9 ± 27.2 | 4.7 ± 1.7 | 5.3 ± 1.7 | - | - |
| Lund | PM 2015 | 8.1 ± 2.6 | 9 ± 2.3 | 23 ± 11.6 | 25.5 ± 10.5 | 9.8 ± 5.3 | 12.1 ± 6.3 | 45.4 ± 23.1 | 47.3 ± 17.8 |
| Uppsala | PF 2014 | 5.8 ± 1.5 | 8.2 ± 3.9 | 12.8 ± 5.2 | 15.5 ± 7 | 8.4 ± 2.8 | 8.8 ± 3.2 | - | - |
| Uppsala | PF 2015 | 31.6 ± 9.7 | 30.2 ± 12 | 60.1 ± 25 | 56.5 ± 29.7 | 4.3 ± 3.1 | 3.3 ± 2.7 | - | - |
| Uppsala | PM 2015 | 7.4 ± 2.8 | 9 ± 3.2 | 19.4 ± 6.9 | 28.5 ± 16.7 | 6.6 ± 3.2 | 5 ± 4.2 | 37.4 ± 17.1 | 36.9 ± 17.9 |
| Umeå | PF 2014 | 10.7 ± 7.8 | 12.6 ± 5.6 | 12.8 ± 13.8 | 19 ± 14.1 | 4.1 ± 1.8 | 4.9 ± 2.3 | - | - |
| Umeå | PF 2015 | 17.3 ± 8.6 | 22.3 ± 9.7 | 39.7 ± 34.4 | 53.5 ± 36.4 | 3.8 ± 1.9 | 4.6 ± 2.2 | - | - |
| Umeå | PM 2015 | 7.8 ± 5.2 | 9.6 ± 4.4 | 28.2 ± 28.4 | 25.3 ± 26.9 | 4.3 ± 2.7 | 5.1 ± 2.3 | 37 ± 25.3 | 36.1 ± 14.6 |
| Kiruna | PF 2014 | 14.6 ± 4.3 | 17.9 ± 4.3 | 36.4 ± 27 | 38.3 ± 23.7 | 7.2 ± 2.7 | 7.9 ± 3.5 | - | - |
| Kiruna | PF 2015 | 14.6 ± 4.4 | 18.9 ± 5.7 | 32.8 ± 23.4 | 41.1 ± 24.8 | 6.7 ± 2.5 | 7 ± 3.4 | - | - |
| Kiruna | PM 2015 | 7.8 ± 2.1 | 11.7 ± 3.1 | 11.6 ± 6.1 | 23.1 ± 10.9 | 8.7 ± 3.4 | 12.8 ± 8.2 | 46.5 ± 19.4 | 52.5 ± 16.6 |
|  |  | Flower number ± SD | | Fruit number ± SD | | Clone number ± SD | |  |  |
| Region | Time / Year | ambient | elevated | ambient | elevated | ambient | elevated |  |  |
| Lund | PF 2014 | 0 ± 0 | 0 ± 0 | - | - | - | - |  |  |
| Lund | PF 2015 | 4.5 ± 4.1 | 6.2 ± 4.6 | 0.4 ± 0.8 | 1.1 ± 1.4 | - | - |  |  |
| Lund | PM 2015 | - | - | - | - | 0.6 ± 0.7 | 0.9 ± 0.8 |  |  |
| Uppsala | PF 2014 | 3.5 ± 2.4 | 4.2 ± 3.3 | - | - | - | - |  |  |
| Uppsala | PF 2015 | 2 ± 2.3 | 1.5 ± 2.6 | 0.9 ± 2.3 | 0.8 ± 1.8 | - | - |  |  |
| Uppsala | PM 2015 | - | - | - | - | 0.5 ± 0.6 | 0.4 ± 0.7 |  |  |
| Umeå | PF 2014 | 0.6 ± 1.1 | 0.8 ± 1.2 | - | - | - | - |  |  |
| Umeå | PF 2015 | 1 ± 1.8 | 1.4 ± 1.9 | 0.3 ± 0.8 | 0.6 ± 1.1 | - | - |  |  |
| Umeå | PM 2015 | - | - | - | - | 0.4 ± 0.5 | 0.2 ± 0.4 |  |  |
| Kiruna | PF 2014 | 1.4 ± 2.5 | 1.9 ± 4.2 | - | - | - | - |  |  |
| Kiruna | PF 2015 | 4.2 ± 4.4 | 10.9 ± 10.1 | 3.1 ± 2.6 | 3 ± 2.9 | - | - |  |  |
| Kiruna | PM 2015 | - | - | - | - | 0.5 ± 0.7 | 0.8 ± 0.7 |  |  |
